# Establishment of a novel extracorporeal bowel model to study luminal approaches to treat inflammatory bowel disease

**DOI:** 10.1242/dmm.011734

**Published:** 2013-08-14

**Authors:** Anne Breitrück, Gisela Sparmann, Steffen Mitzner, Claus Kerkhoff

**Affiliations:** 1 Division of Gastroenterology, Department of Medicine II, University of Rostock, 18057 Rostock, Germany; 2 Fraunhofer Institute for Cell Therapy and Immunology, Department of Immunology, Project group “Extracorporal Immunomodulation”, 18057 Rostock, Germany; 3 Division of Nephrology, Department of Medicine II, University of Rostock, 18057 Rostock, Germany

## Abstract

We have established an extracorporeal bowel model system for the analysis of early events in inflammatory bowel disease (IBD) and therapeutic applications. This model consists of an intestinal segment that is cannulated and perfused *in situ*, allowing the investigation of cellular responses of apical mucosa cells on luminal applied substances. Short-term treatment with iodoacetamide mimicked experimental intestinal inflammation in IBD, as indicated by histological alterations such as hemorrhage, hyperemia and loss of regular crypt architecture, as well as enhanced expression of cytokines (e.g. IL-6, IL-10 and MCP-1) compared with control segments perfused with media. Perfusion of therapeutic agents (e.g. dexamethasone or Mutaflor) in the small intestine segment significantly reduced the features of early inflammation that are induced by iodoacetamide. Moreover, similar data were obtained for Resormin^®^, a montmorillonite-illite mixed-layer mineral (smectite), indicating that smectites might be a newly identified therapeutic option for IBD. In summary, this model could provide novel insights into epithelial injury as well as genesis of IBD and, therefore, be useful in testing the therapeutic potential of compounds for IBD therapy.

## INTRODUCTION

Crohn’s disease (CD) and ulcerative colitis (UC), collectively termed inflammatory bowel diseases (IBD), represent chronic relapsing and remitting disorders of the gastrointestinal tract. Recent evidence indicates that IBD is a result of a genetic predisposition that leads to a mucosal immune regulatory cell defect, barrier defects and susceptibility to environmental triggers, including luminal bacteria and specific antigens (Seksik et al., 2006; Scaldaferri and Fiocchi, 2007; Podolsky, 2002; Schreiber et al., 1992). Although researchers have long recognized this complex interaction among genetics, the immune system and the environment, the understanding of the contribution of each of these factors to IBD pathogenesis continues to deepen.

Several IBD therapies have been established, generally targeting events downstream of the inflammatory cascade (Girardin et al., 2012; Pithadia and Jain, 2011). However, none of them is disease specific and there is currently no cure for IBD. Thus, people have to endure lifelong drug treatment and/or surgical care.

Multiple animal models of IBD have been established; in general, these models can be mainly categorized into five different groups: (1) antigen-specific and bacterial models; (2) other inducible models (chemical, immunological and physical); (3) genetic models (both transgenic and knockout); (4) adoptive transfer models; and (5) spontaneous models (Hoffmann et al., 2002). Although none of these models completely reproduces human IBD, animal models of intestinal inflammation have provided useful insight into the pathogenesis of the intestinal inflammatory response.

The most common chemicals used in induced models causing acute destruction of the intestinal barrier are dextran sodium sulphate (DSS) (Okayasu et al., 1990), trinitrobenzene sulphonic acid (TNBS) (Neurath et al., 1995) and oxazolone (Boirivant et al., 1998). These models have advantages and disadvantages that have been extensively reviewed by Hoffmann et al. (Hoffmann et al., 2002). One major disadvantage, however, is that the onset of intestinal inflammation takes 1–2 weeks.

Our approach has been to establish an extracorporeal bowel model system that allows the analysis of early events in experimental intestinal inflammation and investigation of the suitability of compounds for IBD therapy. The single-pass intestinal *in situ* perfusion method was used, and an experimental design was chosen that warranted vitality and functionality of the perfused segment during the experiment. The mucosal injury was induced by the sulphydryl blocker iodoacetamide (IA). IA is an alkylating agent that reacts with cellular proteins and endogenous glutathione, thereby inducing irreversible mucosal cell damage. The application of sulfhydryl blockers as chemical inducers of inflammation has several advantages: increased vascular permeability, massive mucosal edema, erosion and ulcers become visible within several hours followed by acute inflammation in colon and small intestine (Rachmilewitz et al., 1997; Satoh et al., 1997; Tolstanova et al., 2012). This enabled us to demonstrate that short-term *in situ* perfusion of IA for 15 minutes followed by perfusion of either control media [Dulbecco’s modified Eagle’s medium (DMEM)] or drugs (e.g. dexamethasone and Mutaflor) for 2 hours mimicked both some early events in intestinal inflammation and mucosal healing after IBD therapy. Furthermore, we found that treatment with Resormin^®^, a montmorillonite-illite mixed-layer mineral (smectite) from Friedland (Germany), has similar beneficial effects in the extracorporeal bowel model system. Our model might therefore be useful in the analysis of the pathogenesis of mucosal injury as well as in providing insight into the therapeutic potential of novel compounds.

**RESOURCE IMPACT**

**Background**

Crohn’s disease and ulcerative colitis are types of inflammatory bowel disease (IBD). Both are chronic, non-infectious inflammatory diseases of the human gastrointestinal tract. Although there is growing evidence that a combination of intestinal barrier dysfunction with an overactive immune system plays a key role in IBD, the etiopathogenesis of this group of diseases still remains largely unknown. Moreover, there is currently no cure for IBD, and affected individuals usually have to undergo lifelong drug therapy and/or surgical treatment. Several animal models have been established, allowing the analysis of the pathology of IBD. However, one major disadvantage of these models is the length of time required for experimental intestinal inflammation to develop. Therefore, the aim of the present study was to establish an extracorporeal bowel model system that allows both the analysis of early events in experimental intestinal inflammation and the testing of compounds that could potentially be used to treat IBD.

**Results**

The authors generated an extracorporeal bowel system based on a single-pass intestinal *in situ* perfusion method, using iodoacetamide (IA), an alkylating agent that induces irreversible mucosal cell damage. Short-term *in situ* perfusion of IA induced significant mucosal damage and severe inflammation. Histological examination of intestinal segments revealed histological alterations, such as hemorrhage, hyperemia and loss of regular crypt architecture. IA treatment also significantly enhanced mRNA transcript levels of pro-inflammatory cytokines and reduced the mRNA expression of the mucosal defense factor intestinal alkaline phosphatase (iAP). These data indicate that IA treatment mimicked the experimental intestinal inflammation characteristics of IBD. Interestingly, the IA-induced mucosal damage was markedly reversed by a consecutive perfusion of the therapeutic agents dexamethasone or Mutaflor. Moreover, similar beneficial effects were obtained with Resormin^®^, a montmorillonite-illite mixed-layer mineral (smectite). Therefore, treatment with a smectite could be a novel therapeutic option for IBD.

**Implications and future directions**

Over the last decade, several IBD therapies have been established; however, none of these therapies is specifically targeted to IBD. A key challenge is the development of a tailored approach to prevent the initiation and perpetuation of the inflammatory cascade before tissue injury occurs. The present extracorporeal bowel model system represents a novel tool for the analysis of the underlying molecular mechanisms as well as for the therapy of IBD; thus, the system has implications for both basic researchers and clinicians.

## RESULTS

### Effects of IA and therapeutics on the apical and basal mucosa of small intestine segments

**Fig. 1.**
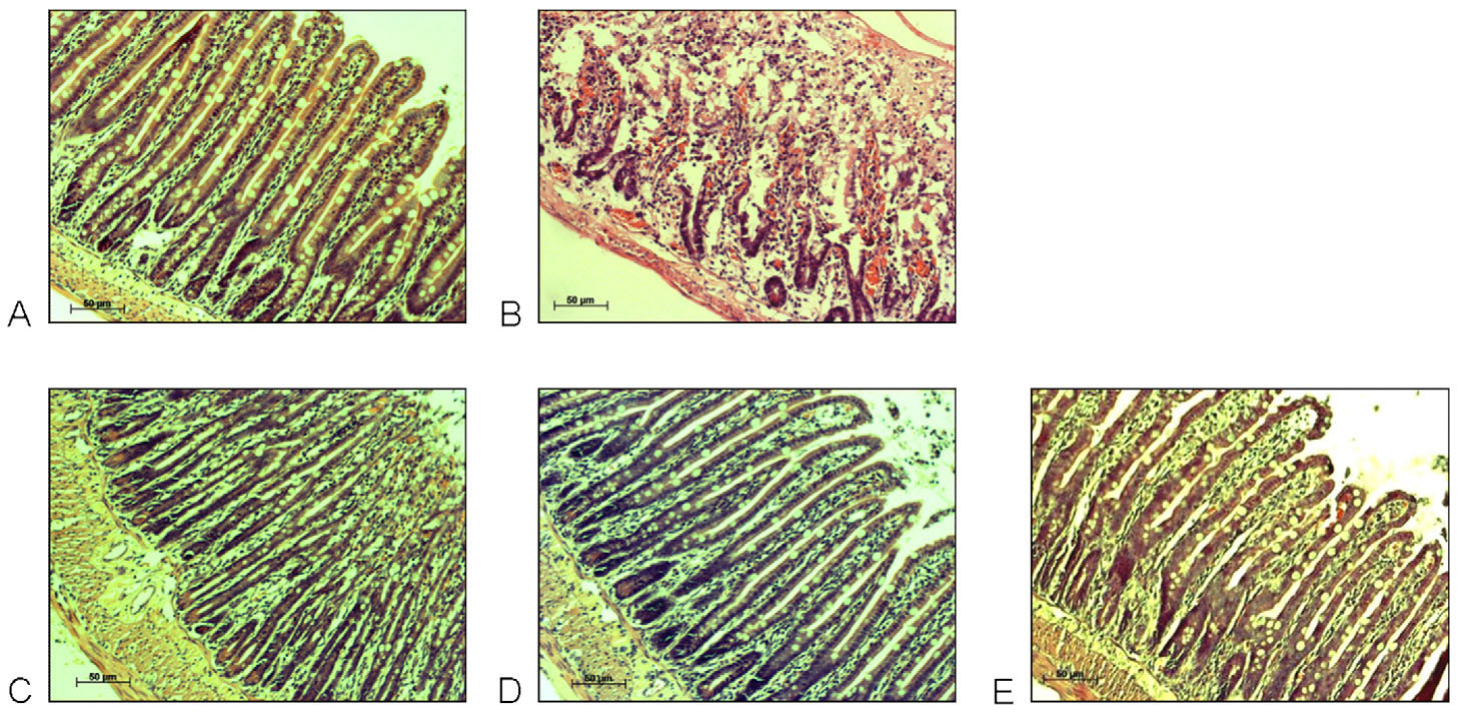
**Representative histology pictures from H&E-stained intestinal segments *in situ* perfused with various therapeutics.** Samples were perfused with: DMEM (A), IA + DMEM (B), IA + Mutaflor (C), IA + dexamethasone (D) or IA + Resormin^®^ (E). Original magnification: 100×. (A) A relatively normal histological appearance is evident in the small intestine perfused with DMEM alone. (B) Evidence of crypt damage, submucosal edema and necrosis in the lamina propria and sub-mucosa is present in the small intestine after perfusion with IA + DMEM. (C–E) Reduced pathology is observed in the small intestine *in situ* perfused either with IA + Mutaflor (C), IA + dexamethasone (D) or IA + Resormin^®^ (E).

Short-term *in situ* perfusion of IA for 15 minutes followed by perfusion of DMEM for 2 hours induced significant mucosal damage and signs of severe inflammation. Histological examination showed loss of regular crypt architecture, large cellular debris and enhanced infiltration of neutrophils compared with untreated segments (Fig. 1B). In the control group, the animals were treated similarly to the therapy groups, with the exception that the jejunum was solely perfused with DMEM. The histological examination of the control animals showed normal architecture of the mucosal and submucosal components (Fig. 1A), excluding the possibility that the action of the peristaltic pump itself had caused mucosal damage.

In the case of IA treatment followed by perfusion of the therapeutic agents Mutaflor, a probiotic consisting of a viable nonpathogenic bacterial strain (*Escherichia coli* Nissle 1917), or the glucocorticoid dexamethasone, the mucosal damage that was induced by IA was markedly reversed. There was apparently normal mucosal architecture after 2 hours (Fig. 1C,D). Resormin^®^ is a montmorillonite-illite mixed-layer mineral (smectite) from Friedland, Germany. Smectites have been used in the treatment of acute diarrhea in adults and children (Khediri et al., 2011; Szajewska et al., 2006). Furthermore, the use of smectites for the relief of reflux (Gouyon et al., 1989) diseases as well as the support of gut renovation has been reported (Gardiner et al., 1993). In accordance with this latter study, IA treatment followed by perfusion with Resormin^®^ improved IA-induced mucosal injury and reconstituted normal mucosal architecture (Fig. 1E). Clearly, the extracorporeal bowel model system would prove a practical method of testing the therapeutic potential of these compounds.

**Fig. 2.**
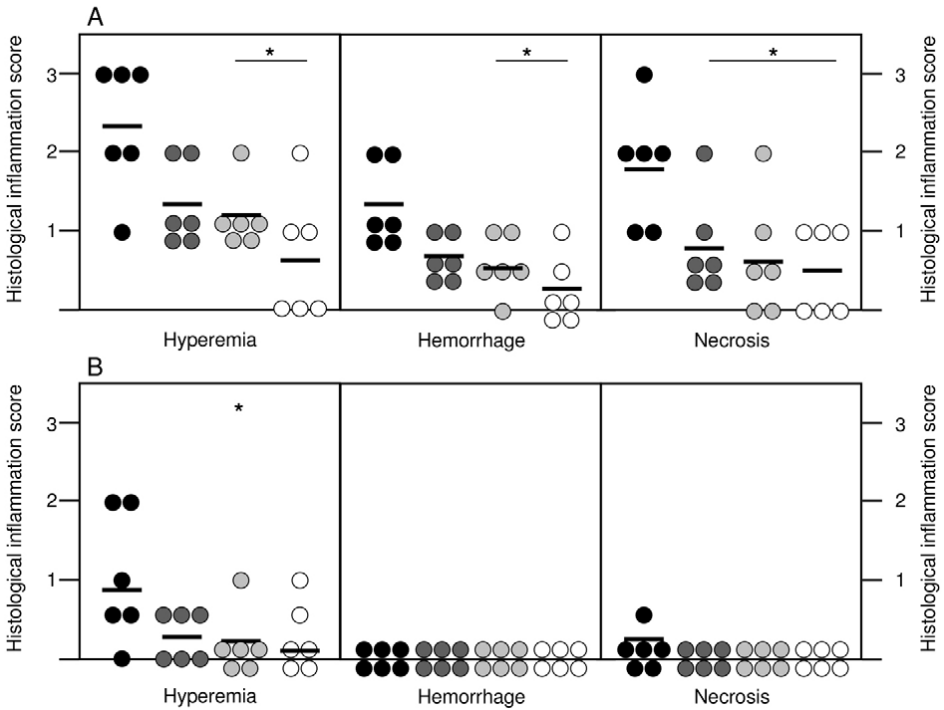
**Comparison of histological scores for hyperemia, hemorrhages and necrosis in the apical and basal mucosa of the four different experimental groups**. (A) Apical mucosa; (B) basal mucosa. Each symbol represents an individual rat (*n*=6/group), and the mean values of the experimental groups are indicated. Morphological changes were assessed by the modified histological scoring system described in Table 1. *P<0.05 versus IA. Black, IA; dark gray, IA + Mut; light gray, IA + Dex; white, IA + Res.

The extent and severity of mucosal inflammation was assessed by histological scoring, which provided detailed assessment of subjective and objective signs and symptoms of inflammation. Regions of hyperemia, hemorrhages and necrosis, both in apical and basal mucosa, were analyzed by a pathologist in a blinded fashion. Each parameter was scored from 0 to 3 depending on the severity of inflammation, with 0 being no activity and 3 considered severe activity. Therefore, combining the scores from both regions, the maximal value of the score that could be achieved was 18. Significant mucosal damage and severe inflammation was noted in the apical mucosa in particular (Fig. 2). The histological score confirmed that the three groups treated with IA followed by perfusion with dexamethasone, Mutaflor or Resormin^®^ had significantly reduced signs and symptoms of inflammation compared with controls.

**Fig. 3.**
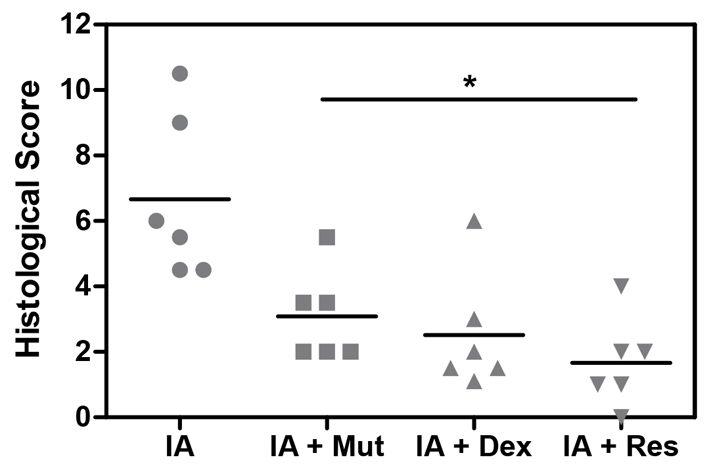
**Summary of the intestinal histological score data in the four different experimental groups**. Bars indicate median. *P<0.05 versus IA.

The overall histological score of animals treated with IA, IA + Mutaflor or IA + dexamethasone was estimated at 6.58±0.96, 3.08±0.57, and 2.58±0.72, respectively (Fig. 3). The values indicate the beneficial effects of the drugs in reversing the IA-induced mucosal damage. Interestingly, Resormin^®^ exerted the strongest beneficial effect. The overall histological score of IA + Resormin^®^-treated animals was estimated to 1.67±0.71.

Immune cell infiltration into the perfused intestinal segments after IA treatment alone or in combination with dexamethasone, Mutaflor or Resormin^®^ was analyzed immunohistochemically. No significant differences in the recruited cell populations between the different groups were seen for CD4^+^, CD8^+^, αβ TCR^+^ or γδ TCR^+^ cells. However, a reduced number of CD68^+^ cells was present after perfusion of the therapeutic agents compared with in IA-treated control animals, although this was not significant.

### Effects of iodoacetamide and therapeutics on the induction of cytokines

**Fig. 4.**
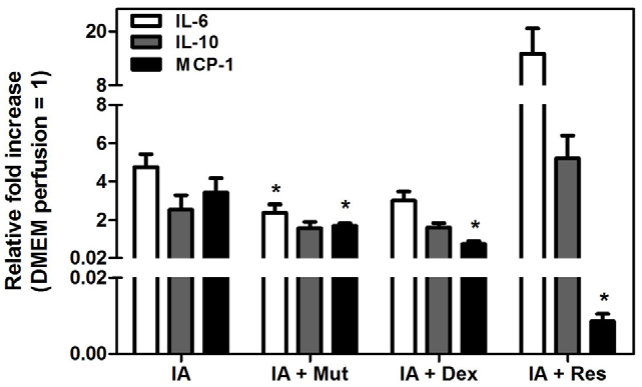
**Quantitative RT-PCR analysis of pro-inflammatory cytokines in the perfused intestinal segments of the four treatment groups**. The values are expressed as mean ± s.e.m. of mRNA levels (*n*=6 per group) compared with intestinal segments perfused with DMEM (pooled samples). *P<0.05 versus IA.

Cytokines play a key role in IBD by affecting the recruitment, activation and differentiation of various subsets of T cells. Their levels in time and space orchestrate the development, recurrence and exacerbation of the inflammatory process in IBD (Zhong et al., 2008). We therefore analyzed the expression of several cytokines after IA treatment alone or in combination with dexamethasone or Mutaflor. IA treatment significantly enhanced the levels of mRNA transcripts of *IL-6*, *IL-10* and *MCP-1* compared with those seen in control segments perfused with DMEM. Treatment with either dexamethasone or Mutaflor significantly reduced the IA-induced expression of *IL-6*, *IL-10* and *MCP-1* compared with levels in IA-treated controls (Fig. 4). The actions of Resormin^®^ were more complex. Treatment with IA followed by perfusion of Resormin^®^ strongly reduced the gene expression of *MCP-1* but enhanced the gene expression of *IL-10* and *IL-6*.

### Expression of intestinal alkaline phosphatase

The brush border enzyme intestinal alkaline phosphatase (iAP) is expressed mainly in the small intestine, acting as a mucosal defense via lipopolysaccharide (LPS) dephosphorylation (Poelstra et al., 1997; Bates et al., 2007). In individuals with IBD, iAP expression is strongly reduced in inflamed tissue compared with non-inflamed tissue samples (Tuin et al., 2009), indicating that it can serve as an early marker of intestinal inflammation. iAP was therefore measured in the extracorporeal bowel model system.

**Fig. 5.**
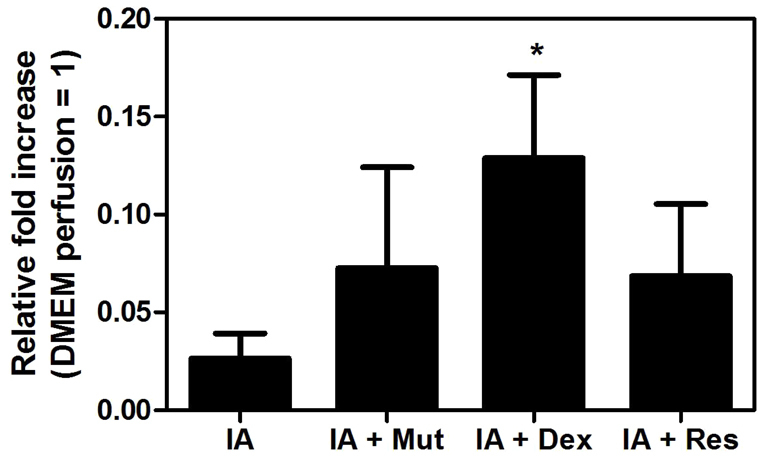
**Quantitative RT-PCR analysis of *iAP* in the perfused intestinal segments of the four treatment groups.** The values are expressed as mean ± s.e.m. of mRNA levels (*n*=6 per group) compared with intestinal segments perfused with DMEM (pooled samples). *P<0.05 versus IA.

Expression of the *iAP* gene was strongly suppressed in the intestinal segments after IA treatment, whereas treatment with either dexamethasone, Mutaflor or Resormin^®^ resulted in increased transcript levels compared with IA treatment alone. It is noteworthy that dexamethasone was the most effective therapeutic out of those tested (Fig. 5).

## DISCUSSION

Short-term treatment of an extracorporeal bowel model system with IA mimicked in some aspects the intestinal inflammation seen in IBD. In accordance with Satoh et al. (Satoh et al., 1997), a single treatment with IA was sufficient to induce the characteristic features of inflammation caused by chemically induced mucosal damage (Rachmilewitz et al., 1997). The IA-treated segments showed histological alterations, including hemorrhage, hyperemia and loss of crypt architecture. There were increased levels of pro-inflammatory cytokines, e.g. IL-6 and MCP-1, accompanied with enhanced levels of the anti-inflammatory cytokine IL-10. Moreover, IA-treated segments had decreased iAP, an early marker of intestinal inflammation (Tuin et al., 2009). In addition, we noted a trend, although not significant, of enhanced CD68^+^ cell infiltration.

The IA-induced inflammatory response bears similarity to the well-established dextran sodium sulfate (DSS)-induced colitis model, which chemically induces mucosal cell damage. The main advantage of IA is the fact that the mucosal injury becomes visible within hours (Satoh et al., 1997; Rachmilewitz et al., 1997). In contrast, the continuous administration of DSS in drinking water for 5–7 days induces acute colitis, and the earliest histological alterations are detectable after 3 days (Cooper et al., 1993). However, the single-pass intestinal *in situ* perfusion model seems to be applicable to the study of mechanisms of epithelial injury rather than to explore the immune-cell-mediated responses.

It is conceivable to modify the single-pass intestinal *in situ* perfusion model in many ways: one possibility might be the extension of treatment, i.e. short-term application of IA followed by sequential perfusion of DMEM or a therapeutic agent over several hours. Under these conditions, an enhanced immune cell infiltration might then become detectable. Another possibility is the analysis of different segments of the gut, such as the jejunum, ileum and colon. This might be of interest if the scientific and therapeutic focus is directed towards CD and UC.

The extracorporeal bowel model has also been useful in exploring both recovery and analysis of the potential of compounds for IBD therapy. Dexamethasone and Mutaflor are well known for their therapeutic potency in IBD therapy (Irving et al., 2007; Bossa et al., 2009; Boudeau et al., 2003). Consistent with data from these previous studies, both dexamethasone and Mutaflor were found to ameliorate IA-induced intestinal inflammation in the present study. After 2 hours of treatment, the characteristic signs and symptoms of inflammation, such as loss of regular crypt architecture, enhanced levels of cytokines and reduced *iAP* gene expression, were almost absent in intestinal segments after IA treatment. These data show that the model is useful in analyzing early events of intestinal inflammation by exploring cellular responses of apical mucosa cells on luminal-applied substances and, therefore, that it might be an excellent tool for investigating the potential of new therapeutic agents. A short duration of *in situ* perfusion might also allow prompt analysis of the perfused segments, thereby providing an outline of therapeutic potential within several hours. Furthermore, it might enable the evaluation of therapeutic agents concerning their anti-inflammatory potential and the necessity of long-term (*in vivo*) experiments.

The single-pass intestinal *in situ* perfusion method is commonly used either to investigate water and electrolyte absorption in the intestine or to determine the intestinal mechanism of the absorption of drugs (Salphati et al., 2001; Cook and Shenoy, 2003; Kreydiyyeh et al., 2006; Nagare et al., 2010). It has also been applied in experimental IBD models for studying the impact of barrier and transport function, as well as the inhibitory effect on fluid absorption (Stein et al., 1998; Mourad et al., 2003). However, the present study seems to represent the first report that experimental inflammation could be induced by single-pass *in situ* perfusion with IA. This extracorporeal bowel model could be useful for studying the interaction of recruited and activated regulatory immune cells with damaged apical mucosa cells and commensal microflora.

Finally, our model could be of importance in testing the therapeutic potential of novel compounds for IBD therapy. We found that a montmorillonite-illite mixed-layer mineral (smectite) from Friedland referred to as Resormin^®^ exerted beneficial effects after IA-induced mucosal injury. Compared with the established therapeutic agents dexamethasone and Mutaflor, Resormin^®^ displayed the strongest reduction of the overall histological score after treatment with a combination of IA and smectite. Furthermore, it strongly suppressed the IA-induced gene expression of *MCP-1* but enhanced the expression of *IL-10* and *IL-6*.

Numerous studies have investigated the anti-inflammatory effects of IL-10 and suggested the importance of its dysregulation in different entities. For example, overexpression has been found in lymphoma, whereas IL-10 deficiency was found in IBD, indicating that the dysregulation of IL-10 has a pathophysiological significance (Asadullah et al., 2003). Similarly, altered IL-6 production has been found in inflammatory states such as rheumatoid arthritis, CD and UC. Although the diversity of IL-6-dependent effects is extremely widespread, IL-6 has a protective effect during recovery from DSS-colitis because it promotes the survival of intestinal epithelial cells (Grivennikov et al., 2009; Jin et al., 2010). The reduced production of MCP-1 in combination with the enhanced expression of IL-10 and IL-6 seen in IA + Resormin^®^-treated animals might provide an additional mechanistic explanation for the beneficial effects of the smectite in acute mucosal injury.

Montmorillonite-illite mixed-layer minerals are three-layer minerals. A special feature of these minerals is the small particle size, resulting in large surface areas as well as a strong ion-exchange capacity, which is determined by its mineralogical and/or chemical composition (Kaufhold and Dohrmann, 2008). Because of these properties, montmorillonite-illite mixed-layer minerals have been used for decades as a pharmaceutical excipient and as a bioactive agent in both animal husbandry and humans. In addition, they have been applied for the relief of diarrhea and reflux diseases (Gouyon et al., 1989; Szajewska et al., 2006; Khediri et al., 2011) as well as the support of gut renovation (Gardiner et al., 1993). It is worthwhile mentioning that anti-inflammatory potential has been demonstrated for another mixed-layer mineral: diosmectite reduces the degree of inflammation in TNBS colitis (Gardiner et al., 1993; González et al., 2004), decreases ionic conductance as well as mannitol and horseradish peroxidase permeability in TNFα-stimulated epithelial cells (Mahraoui et al., 1997), and suppresses the LPS-induced IL-8 and IL-1β secretion from human intestinal epithelial cells and THP-1 cells (González et al., 2004). However, the underlying molecular mechanisms by which Resormin^®^ exerted the beneficial effects in acute mucosal injury remain unclear and will be the objective of a prospective study.

In summary, our extracorporeal bowel model could provide novel insights into the underlying mechanisms of epithelial injury as well as the genesis of IBD and, therefore, be useful in testing the therapeutic potential of compounds for IBD therapy.

## MATERIALS AND METHODS

### Animals

Lewis rats (male, 170–200 g) obtained from Charles River Laboratories (Sulzfeld, Germany) were housed under controlled environmental conditions regarding temperature and humidity with a 12:12 hour light:dark cycle. They were given standard food and water *ad libitum*. The experiments were done under the German protection law, and were approved by the local animal care and use committee (2009/02/27; LALLF M-V/TSD/7221.3-1.1-065/08).

### *In situ* perfusion

**Fig. 6.**
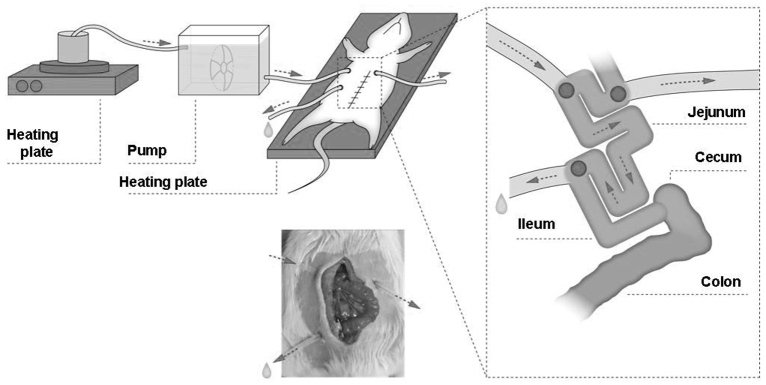
**Schematic diagram of the extracorporeal bowel model system**. See text for details.

Prior to *in situ* perfusion, the rats were fasted for 12 hours, but had free access to water. They were anesthetized by an intraperitoneal injection of pentobarbital at 50 mg/kg body weight. To maintain the body temperature during the perfusion, the rats were placed on a temperature-controlled operating table. The abdomen was opened by a midline incision and an approximately 6 cm segment of the jejunum was identified. This intestinal segment was not separated from the mesentery or afferent and efferent blood vessels, allowing maintenance of vitality of the segment during the experiment. A single semicircular incision was made at each end of the segment and two polythene tubes (1.5 mm ID, 2.7 mm OD) used for cannulation were then fixed proximally and distally by ligation. To avoid intestinal obstruction by the luminal contents, a third cannula was placed ∼2 cm proximal from the intestinal segment in the upper jejunum. The intestine was returned to the abdominal cavity and the abdomen was closed by suture (Fig. 6). The proximal cannula was connected to a peristaltic pump so that the intestinal segment could be perfused *in situ* at 0.1 ml/minute with normal saline (37°C) until the fluid appears clear. The flow of the perfusate in this experiment was a single pass. Local damage in the isolated intestine was induced by 15 minutes perfusion with 1% IA dissolved in 1% methylcellulose, followed by 2 hours perfusion with DMEM.

In the therapy approach, dexamethasone (1 mg/kg body weight dissolved in DMEM) or Mutaflor (3×10^8^ CFU) were perfused for 2 hours after IA treatment. The montmorillonite-illite mixed-layer mineral (smectite) referred to as Resormin^®^ was provided by FIM Biotech, Friedland, Germany. Resormin^®^ was suspended as 10% suspension in DMEM and also perfused for 2 hours after IA treatment. In control experiments, we did not observe any effect on mucosal architecture if Resormin^®^ was applied alone.

At the end of the experiments, the animals were killed by cardiac punctuation. The perfused intestinal segment was removed and cut in two; one part was stored in liquid nitrogen for RNA isolation, and the other part was unrolled and divided longitudinally into two further parts. One half of this was fixed and embedded in paraffin for hematoxylin and eosin (H&E) staining. The other half was embedded in Tissue Tek^®^ and snap-frozen.

### Quantitative reverse transcriptase PCR using real-time TaqMan™ technology

Total RNA from biopsies of the perfused segment was isolated with TRIzol^®^ reagent. A sample of 0.25 μg RNA was reversed transcribed into cDNA using oligo (dt) 12–18 primer and superscript II reverse transcriptase (Sparmann et al., 2010). Relative quantification of target cDNA levels by real-time PCR involved an API Prism 7000 sequence detection system (Applied Biosystems, Foster City, CA). TaqMan^®^ universal Master Mix and the following rat-gene-specific assays on Demand™ were used: Rn 00561420_m1 (IL-6), Rn 00563409_m1 (IL-10), Rn 00580555_m1 (MCP-1), Rn 01527840_m1 [hypoxanthinephosphoribosyltransferase (HPRT); housekeeping gene control]. A custom plus TaqMan^®^ RNA assay was used for the *iAP* gene expression. The cycler conditions were 10 minutes at 95°C, followed by 50 cycles of 15 seconds at 95°C and 1 minute at 60°C. PCR reactions were performed in triplicates. The relative expression of each mRNA was compared with HPRT (ΔCt=Ct_target_ − Ct_HPRT_). The relative amount of mRNA in the perfused segment was expressed as 2−ΔΔct, where ΔΔct represents the difference between Ct from the DMEM control perfusion (pooled samples) and each sample = Δct_perfused_ − Δct_control perfusion DMEM_.

### Microscopic assessment and histology

After fixing and embedding of the tissue in paraffin, thin sections were cut and stained with H&E using standard methods. Pathological changes in the sections of the intestinal segments were assessed using a modified scoring procedure (Satoh et al., 1997). With regard to short application of IA, our grading system was based on the alterations seen in the apical and basal mucosa, such as hyperemia, hemorrhages and necrosis. These were assessed by a pathologist in a blinded fashion. The grade was assigned a number based on the degree of inflammation, which was usually scored from 0 to 3, 0 being no activity and 3 severe activity. The maximal value of the score that could be achieved was 18 (Table 1).

**Table 1.**
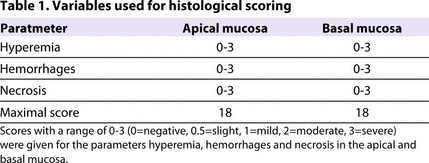
Variables used for histological scoring

### Immunohistochemistry

Cryostat sections (5 μm) were air dried and fixed in ice-cold methanol for 3 minutes. The following incubation steps were carried out in a humid chamber. Fixed sections were first incubated for 5 minutes in 35% H_2_O_2_ to block endogenous peroxidase, followed by incubation with Avidin D solution for 15 minutes and biotin solution for 15 minutes (Avidin/Biotin blocking kit, Vector Laboratories, Burlingame, CA). The slides were incubated with blocking serum (PBS containing 2% goat serum) for 1 hour followed by exposure to mouse anti-rat antibodies against CD4, CD8, CD68, αβ TCR and γδ TCR, diluted in blocking serum for 1.5 hours. After washing with PBS, the second biotinylated goat anti-mouse IgG antibody was applied for 30 minutes. After washing with PBS, the slides were incubated with a peroxidase-conjugated avidin-biotin system (Vectastain ABC Kit, Vector Laboratories, Burlingame, CA), which was visualized by the peroxidase substrate NovaRed (Vector Laboratories, Burlingame, CA). The slides were counterstained with hematoxylin and mounted in Pertex after dehydration.

### Statistical analysis

Data are expressed as mean ± s.e.m. Statistical analyses were performed using the parameter free Mann-Whitney *U*-test, with a *P*<0.05 being considered significant.
